# Bone and Joint Infections: The Role of Imaging in Tailoring Diagnosis to Improve Patients’ Care

**DOI:** 10.3390/jpm11121317

**Published:** 2021-12-07

**Authors:** Andrea Sambri, Paolo Spinnato, Sara Tedeschi, Eleonora Zamparini, Michele Fiore, Riccardo Zucchini, Claudio Giannini, Emilia Caldari, Amandine Crombé, Pierluigi Viale, Massimiliano De Paolis

**Affiliations:** 1 Orthopedics Unit, IRCCS Azienda Ospedaliero-Universitaria di Bologna, 40138 Bologna, Italy; emilia.caldari@gmail.it (E.C.); Massimiliano.depaolis@aosp.bo.it (M.D.P.); 2 DIMEC, University of Bologna, 40133 Bologna, Italy; sara.tedeschi@unibo.it (S.T.); michele.fiore@ior.it (M.F.); riccardo.zucchini@ior.it (R.Z.); claudio.giannini@ior.it (C.G.); pierluigi.viale@unibo.it (P.V.); 3 Radiology Unit, IRCCS Istituto Ortopedico Rizzoli, 40136 Bologna, Italy; Paolo.spinnato@ior.it; 4 Infectious Disease Unit, Azienda Ospedaliero-Universitaria di Bologna, 40138 Bologna, Italy; Eleonora.zamparini@aosp.bo.it; 5 Department of Oncologic Imaging, Institut Bergonié, Comprehensive Cancer Center of Nouvelle-Aquitaine, 33000 Bordeaux, France; a.crombe@bordeaux.unicancer.fr

**Keywords:** bone infections, prosthesis infections, diagnosis, imaging

## Abstract

Imaging is needed for the diagnosis of bone and joint infections, determining the severity and extent of disease, planning biopsy, and monitoring the response to treatment. Some radiological features are pathognomonic of bone and joint infections for each modality used. However, imaging diagnosis of these infections is challenging because of several overlaps with non-infectious etiologies. Interventional radiology is generally needed to verify the diagnosis and to identify the microorganism involved in the infectious process through imaging-guided biopsy. This narrative review aims to summarize the radiological features of the commonest orthopedic infections, the indications and the limits of different modalities in the diagnostic strategy as well as to outline recent findings that may facilitate diagnosis.

## 1. Introduction

Bone and joint infections (BJI) are a major problem because of important social and financial problems [1,2]. The incidence is assessed to be approximately 70/100,000 patients/year and increases with age [3]. Orthopedic infections represent an extremely heterogeneous group of diseases that require complex medical care, including implant-associated infections (e.g., prosthetic joint infections and infections after fracture fixation), septic arthritis, and osteomyelitis. Numerous operations and long-term antimicrobial treatment are generally necessary to treat infection and restore function.

Imaging is of paramount importance to confirm the diagnosis, establish the gravity and degree of infection, plan biopsy and control the response to therapy. The clinical diagnosis is often uneasy due to the nonspecific symptoms, making imaging crucial to plan patients’ management. Some radiological features are pathognomonic of bone and joint infections for each modality used. However, imaging diagnosis of these infections can be challenging as well, because of several overlaps with non-infectious etiologies. In parallel, these last two decades have shown innovations in quantitative imaging that could provide new clues towards adequate diagnosis, from new contrast media, nuclear tracer to improved imaging post-processing and quantification.

Interventional radiology is generally needed to confirm the diagnosis and to detect the microorganism responsible for the infection through imaging-guided biopsy.

This narrative review aims to summarize the radiological features of the commonest orthopedic infections, the indications and the limits of the different modalities in the diagnostic strategy as well as to recap recent findings that may facilitate diagnosis.

## 2. Acute Osteomyelitis

Osteomyelitis (OM) is a bone inflammation caused by infection. Acute OM can be secondary to hematogenous spread or to direct inoculation by trauma, contiguous or post-operative infection [4]. In the late stages, diagnosis can be easily achieved clinically. However, an early accurate diagnosis is more challenging, and it often necessitates multiple imaging techniques [5].

**Figure 1:**
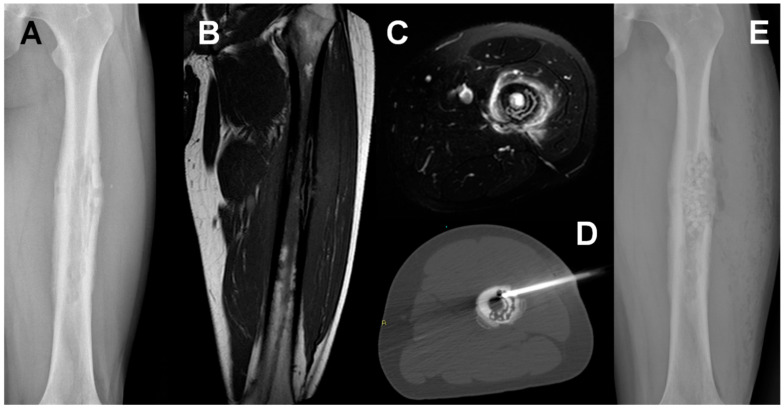
Staphylococcus aureus Osteomyelitis in a 20-year-old man. A conventional radiograph (**A**). MRI coronal T1w (**B**) and axial T2w fat-saturated (**C**) show a permeative lesion of the left femoral shaft. CT-guided biopsy permitted to identify the responsible microorganism (**D**). Conventional radiograph after surgical treatment showed antibiotic microspheres placed into the bone (**E**).

*Radiographs.* Radiographs are the first study indicated when acute OM has been supposed [6,7]. Destruction of cortical bone, permeative marrow lucency, and periosteal reaction can be observed on x-rays in the case of acute OM [8]. Other suggestive signs include joint space widening and soft tissues alterations (swelling, gas, foreign body) (Figure 1A,E). A reduction of 30% to 50% in bone density is required before the radiographic change is apparent. Thus, the sensitivity and specificity of x-rays to detect acute OM and bone findings are relatively low, in particular during the first 10–14 days of infection [9].

*Ultrasound (US).* US represents a non-invasive technique to assess soft tissues and cortical bone; it can guide diagnostic aspiration, drainage, or tissue biopsy. Ultrasound is rapid, low-cost, and does not expose the patient to radiation. However, it largely depends on the operator. Moreover, the permeation and wave reflection can be impeded by gas (intestine) or dense structures (bone), making deep tissue difficult to visualize. US may identify signs of OM earlier than X-rays [10], in particular in children [11]. Periosteal reaction is major in the immature skeleton, principally in long bones [12]. The initial signs of acute OM on the US are juxtacortical swelling of soft tissues and periosteal elevation or thickening. The periosteal abscess must be supposed if a hypo- to hyperechogenic alteration adjacent to the bone surface with adjacent structure dislocation is shown.

*Computed tomography (CT).* CT provides an optimal characterization of cortical bone destruction and periosteal reaction and offers information regarding soft tissue alterations. It is the best technique to detect small foci of gas inside the medullary canal, an uncommon but consistent sign of OM [13] and zones of cortical erosion [14]. It may help to definite the area of the infection, particularly in regions of complex anatomies, such as the spine, and to guide interventional procedures (biopsies and aspirations), particularly in the vertebral column and sacroiliac joints (Figure 1D). Post-contrast CT can help to identify soft tissue abnormalities.

*Magnetic resonance imaging (MRI).* MRI is highly sensitive to detect OM in the first 3–5 days (Figure 1B,C) [15]. Moreover, it provides more accurate information regarding the extent of bone involvement when the diagnosis OM has already been formulated. The most appropriate sequences to detect acute OM are the short tau inversion recovery (STIR) and the T2-weighted imaging (WI) fat-suppressed fast spin-echo (SE) sequences [16]. Edema and exudates within the medullary space produce a low-signal intensity on the T1- weighted images and a high signal on T2 WI and STIR or fat-suppressed sequences. Soft tissues are frequently altered as well, with ill-defined planes. The cortical bone can be interrupted and can have abnormally amplified signal intensity. The absence of cortex thickening helps to differentiate acute from chronic OM [17]. Gadolinium-enhanced sequences help to outline zones of necrosis [15] and are useful to detect abscess [18]. Sinus tracts can extend from the marrow and bone, through the soft tissues, out the skin as high signal intensity spaces on T2-WI [19]. MRI can also help to plan treatment, particularly, percutaneous drainage of fluid collections and surgical debridement. MRI allows to assess the extent of necrotic tissue and to define the dangerous contiguous structures (spine, physes, and joint space), which need customized management to avoid morbidity and complications.

Whole-body (WB)-MRI combines optimal anatomical resolution with the ability to complement the exploration with functional-molecular qualitative and quantitative information via diffusion-weighted imaging (DWI), from nearly the entire body [20,21]. The excellent soft-tissue detail can help in identifying the targets for the collection of microbiological samples from the active areas.

Nonetheless, MRI has several disadvantages. First of all, the acquisition time is long (ranging from 20 to 90 min depending on the machine, sequences protocols, region of interest, and contrast media administration). Thus, patients with a painful disease and/or poor clinical conditions, as well as children, should require other examinations (e.g., CT or PET-CT) or receive sedation/pain therapy before MRI [22,23].

Moreover, MRI is an expensive tool (like PET-CT), and its availability varies depending on geographic areas.

Metal implants should contraindicate MRI because of the presence of ferromagnetic materials, or just reduce the image quality because of metal artifacts. Anyway, several recent improvements (new techniques able to reduce metal artifacts) rendered this issue less problematic [24].

*Nuclear medicine.* The diagnosis of OM can remain doubtful and radionuclide imaging is commonly comprised in the diagnostic work-up. The bone scan is usually positive within 24 to 48 h after the onset of symptoms [25]. Currently, the use of nuclear medicine examination in the diagnostic strategy depends on the pre-test clinical probability for OM. Bone scintigraphy (BS) is helpful to exclude infection when there is a low probability of OM, thanks to its high negative predictive value, especially in a non-operated or recently fractured bone.

*Three-phase BS* (arterial, venous, and bone phases) is typically performed with diphosphonates marked with Technetium-99 m (99 mTc). Their uptake varies on the blood flow, osteoblastic activity, and calcium deposits. OM is diagnosed when there is a focal increase in bone activity in the area of interest on delayed imaging. Furthermore, the positivity on the three phases is highly sensitive for OM (73–100% sensitivity). On the contrary, a normal BS on the three phases almost completely rules out OM due to its high negative predictive value. However, BS lacks specificity (44.6%) and shows overlaps with non-infectious processes (fractures, inflammatory or degenerative osteoarthritis) [26]. Moreover, arterial and venous phases of BS are generally negative in the case of low-grade infection. Increased uptake on the first two phases but not on the third phase can be also observed in patients with soft tissue infection without OM.

*Scintigraphy with gallium citrate (_67_Ga)* can be obtained in combination with a Tc scan. The combined information may be more helpful than each examination alone [27]. The role of _67_Ga scintigraphy is restricted nearly exclusively to the vertebral column.

In patients with a recent fracture or recent surgery, *labeled leukocyte scintigraphy* (LLS) is the first choice [28]. LLS is usually executed with either _111_In oxyquinolone (In) or 99mTc-exametazime. It is less beneficial for infections where the principal cellular response is not neutrophilic (i.e., tuberculosis) [29].

In addition to these traditional radionuclide imaging procedures, positron-emitting radiopharmaceuticals, including fluorodeoxyglucose (FDG) and _68_Ga show promising results [30]. Fusion imaging techniques (combined single-photon emission computed tomography with CT (SPECT/CT) and positron emission tomography with CT (PET/CT)) resulted in significant improvements. These fusion imaging techniques can help in the discrimination between soft tissue and bone infection by providing morphological information. PET is a tomographic technique that allows accurate localization of radiopharmaceutical accumulation. FDG stores in almost all leukocytes and its uptake is associated with their metabolic speed and the number of glucose transporters [31].

The positron-emitting gallium isotope (_68_Ga) has benefits over _67_Ga for diagnosing OM. The half-life of _68_Ga is much shorter than that of _67_Ga (68 min and 78 h, respectively), which allows for the administration of greater amounts of radioactivity. Imaging is executed within a few hours after inoculation, whereas _67_Ga imaging is executed 1–3 days after inoculation [32].

The use of *sodium fluoride* positron emission tomography (18F-NaF PET/CT) has recently been shown to be a useful tool. Following the guidelines from the European Association of Nuclear Medicine (EANM), this tool is indicated in patients with suspected or proved osteomyelitis [33].

However, none of these radiopharmaceuticals is equally efficacious in all body regions. The selection of the appropriate examination should be determined by experts depending on the clinical question and the anatomical setting [30].

Some studies have deemed MRI superior to ^18^F-FDG-PET/CT [34], in particular in zones where detailed structural data [35] or distinction among benign and malignant bone marrow lesions is necessary. Other series observed that the addition of ^18^F-FDG PET/CT to MRI has a specific ability to discriminate degenerative alterations from OM [36]. Hybrid PET/MRI scanners have also been developed, but they are very costly. Thus, it looks well to use MRI as the primary imaging tool for simple cases, whereas PET should be done when there is a (possible) multifocal disease or a contraindication for MRI [37].

## 3. Septic Arthritis

Septic arthritis (SA) is an emergency since it can lead to quick joint destruction and irreversible loss of function within 24–48 h, with a high mortality rate (10–50% in adults).

SA can be hematogenous or come from direct injection of the bacteria in the joint. It can also come from the adjacent spread of OM to the articular surface.

**Figure 2:**
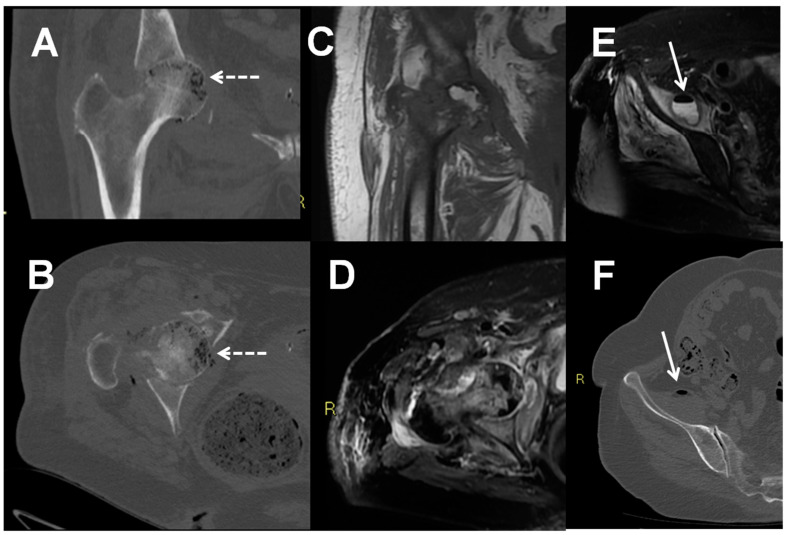
Septic arthritis of the right hip in a 78 years old woman with studied with x-rays (**A**), CT (**B**,**F**), and MRI (**C**,**D**,**E**). CT showed bone intramedullary air coefficients (broken arrows) and involvement of the homolateral ileo-psoas muscle (arrows).

*Radiographs.* Bone erosions, joint space loss, periarticular osteopenia, and soft tissue swelling can be observed on x-rays. In the case of OM, variations in bone signal on both sides of a joint are suggestive of SA. However, radiographs are insensitive, as these signs may be delayed, particularly in non-pyogenic infections. In the beginning, the surrounding soft tissues increase in size, and pseudo widening emerges in the joint interline due to capsule-synovial tumefaction and effusion (Figure 2A).

*Ultrasonography.* US can be helpful to differentiate SA from OM, particularly in the case of hip SA [15]. Lack of a joint effusion has a high negative predictive value for SA [38], while if an effusion is observed it can be either SA or other inflammation of the joint. Power Doppler might help, highlighting the presence of synovial and soft tissue hyperemia [39].

*Computed tomography.* CT is beneficial in particular in SA of fibrocartilaginous joints (pubic symphysis, sacroiliac, or sternoclavicular) and events of concomitant OM (Figure 2B,F).

*Magnetic Resonance Imaging.* MRI is very sensitive to diagnose SA in native hips, with characteristic discoveries of joint effusion, synovial thickening/synovitis, erosions, and periarticular soft-tissue edema [40] (Figure 2C–E). However, specificity can be slightly low, as any inflammatory process of the joint can have an analogous manifestation. MRI findings comprise joint effusion with enhancing synovitis, cartilage thinning, bone erosions, and periarticular soft-tissue edema [41]. Subperiosteal fluid collections can be observed with low signal intensity on the T1-weighted sequences and with intermediate to high signal intensity on the T2 and fat-suppressed images.

*Nuclear Medicine.* Radionuclides studies have a partial role to diagnose SA. It can differentiate OM from soft-tissue infection and detect multifocal joint infections. All three phases of bone scintigraphy show more uptake of the radionuclide due to hyperemia in the synovial vessels [42].

The role of _18_F-FDG PET/CT has not been defined yet [43] because _18_F-FDG accumulates also in inflammatory arthritis, similarly to gallium and labeled leukocytes.

## 4. Chronic Osteomyelitis

Osteomyelitis can be classified based on the onset of symptoms (acute OM within two weeks, subacute OM within one to several months, and chronic OM after a few months). Progression to chronic OM is depicted by periosteal reaction, cortical thickening, and the presence of avascular bony fragments (sequestrum) [44].

**Figure 3:**
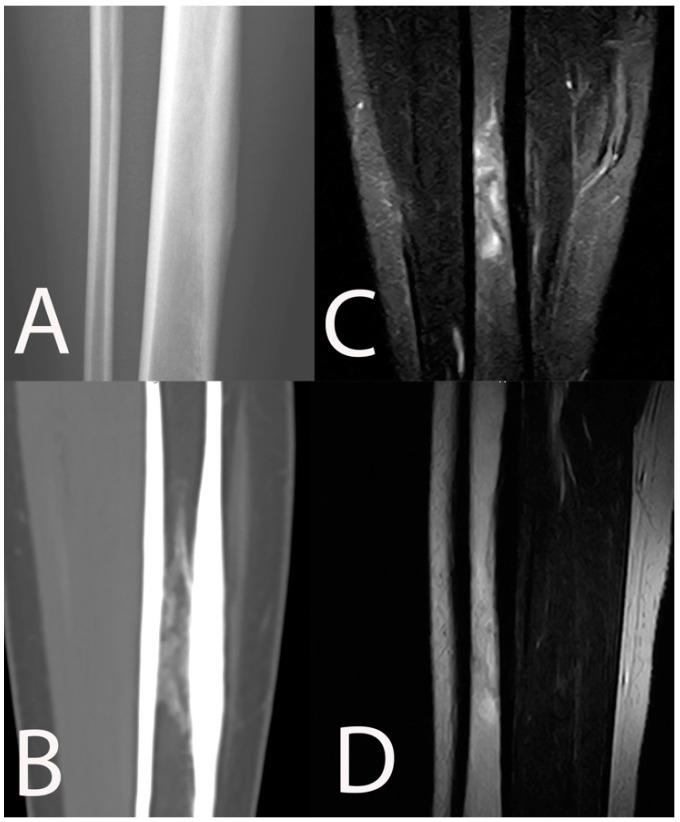
Chronic osteomyelitis of the tibia in a 16-year-old female. Periosteal reaction and sclerotic intramedullary focus are detectable on conventional radiography (**A**) and CT scan (**B**). MRI showed ill-defined bone edema among the sclerotic intramedullary changes on STIR coronal (**C**) and T1w sagittal (**D**).

*Radiographs.* Chronic OM usually appears on x-rays as sclerosis and cortical thickening adjacent to lytic zones within the marrow (Figure 3A) [8]. A lucent sinus tract may be detectable.

*Ultrasonography.* US can aid to evaluate a chronic OM recrudescence that can associate with soft tissue abscess, fistula, or sinus tract [45]. Soft tissue abscess in chronic OM is recognized as a hypo- or anechoic fluid collection.

*Computed tomography.* CT exhibits anomalous inspissation of the affected cortical bone, with sclerosis, invasion of the medullary cavity, and an atypical chronic draining sinus [35] (Figure 3B). The major role of CT in chronic OM is the detection of sequestrum. These fragments of avascular bone can be masked by the adjacent osseous abnormalities on standard X-rays.

Wu et al. [46] recently proposed a machine learning algorithm based on CT scans which exhibited encouraging performances (sensitivity 88.0%, specificity 77.0%) and higher than serum biomarkers such as CRP, ESR, and D-dimer for chronic, post-traumatic OM of the limbs.

*Magnetic resonance imaging.* On MRI, the sequestrum looks like a low-signal area within a granulation tissue inside the bone marrow displaying high signal intensities on T2-WI [47]. Linear high signal intensities on T2-WI which extend across the involucrum correspond to the cloaca, which is a tract leading out of the bone from the medullary cavity. Periostitis can create a border of high signal intensity on T2-WI encompassing the outer surface of the cortex (Figure 3C,D).

Contrast-enhanced T1-WI is essential to localize the sequestrum, as it does not show post-contrast enhancement. The pattern of contrast enhancement can permit the discrimination of fibrovascular scar from infectious foci, facilitating to discern between acute and chronic OM [48].

Squamous cell carcinoma of the sinus tract is an unusual complication of long-lasting chronic OM, which occurs in 0.23% to 1.6%. It can be identified on MRI as an anomalous soft tissue lump [49].

*Nuclear medicine*. PET and SPECT are very precise procedures to assess chronic OM, which allow to differentiate it from soft tissue infections. FDG PET/CT has the highest performances (sensitivity 94%, specificity 87%) to confirm or exclude the diagnosis of chronic OM compared to MRI, BS, or LLS, especially in the axial skeleton [50,51].

## 5. Brodie’s Abscess

Brodie’s abscess is a sub-acute form of OM, frequently with an insidious onset, which displays as a collection of pus in the bone [52]. It is an infection delimited inside the myelum, surrounded by a sclerotic wall, thus minimizing the systemic inflammatory response. It is best detected by the combination of standard x-rays and MRI [53].

*Radiographs.* Brodie’s abscess can have an inconstant look, but it normally looks like a lytic unicameral or multiloculated lesion with a sclerotic rim that is oriented along the long axis of the bone. It is bordered by a thick dense rim of reactive sclerosis that disappears into contiguous bone. A concomitant minor periosteal reaction can be present. The lesion diameter ranges from 4 mm to 5 cm.

**Figure 4:**
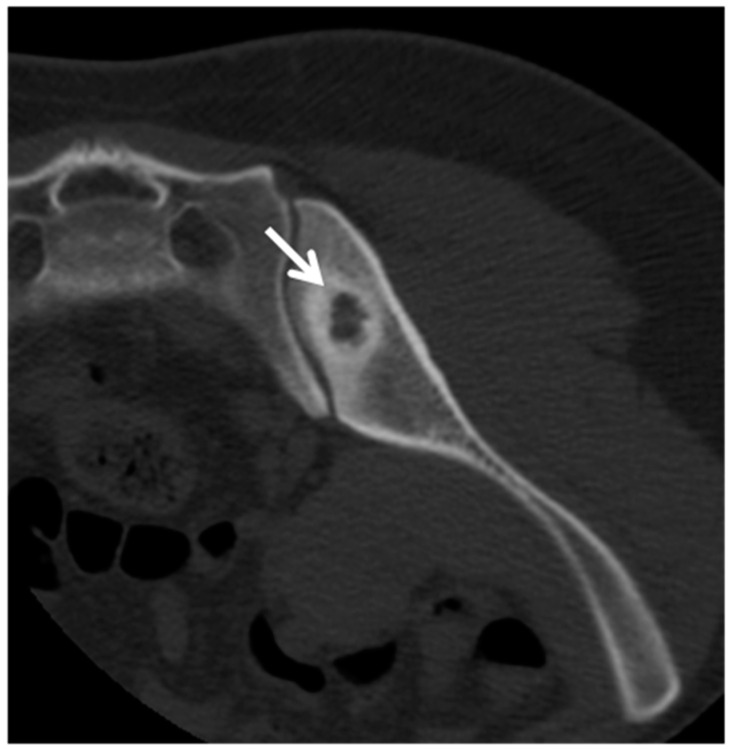
Brodie’s abscess in a 30-year-old man. Computed tomography of the pelvis showed a small (1.5 cm) radiolucent lesion with thick and irregular sclerotic margins (arrow).

*Computed tomography*. Central osteolysis on CT scan with thick rim ossification may be observed, with extensive thick, well-circumscribed periosteal reaction and bone sclerosis around the lesion (Figure 4).

*Magnetic Resonance Imaging.* Brodie’s abscess is visible on MRI as a so-called “target sign”, which is formed by four concentric layers of tissue (necrotic tissue at the center, encircled by an adjacent deposit of granulation tissue, and sclerotic or fibrotic tissue with an outermost rim of edema). Starting centrally and moving outward, this results in T1- weighted sequences in a pattern of low signal (necrosis), intermediate (isointense to muscle) signal (granulation tissue), very low signal (sclerotic or fibrotic tissue), and low signal (edema). On T2 sequences, a pattern of high, intermediate-high, low, and high signal intensities can be appreciated, respectively [54]. Only a peripheral ring enhancement is appreciated after gadolinium administration [55].

*Nuclear Medicine*. Scintigraphy generally shows high activity. Active FDG lesions have been reported in a few reports [56,57]. However, the function of nuclear medicine in the diagnosis of Brodie’s abscess is still debated, and it needs further investigation.

## 6. Diabetic Foot Osteomyelitis

Diabetic foot OM generally comes from contiguous soft tissue ulcers. It is more frequent near the fifth and first metatarsophalangeal joints. The global prevalence of diabetic foot is 6.4% [58]. It can be confused with the rarer Charcot arthropathy (prevalence 0.1%): the damage of osteoarticular structures of a foot on a neuropathic basis but with no infection [59]. In the evaluation of OM, infection focus should be searched close to the ulcer [60].

*Radiographs.* The soft tissues should be assessed for a lucent defect at the skin surface. Foci of air may commonly be found spreading from the ulcer to the infected bone [61]. The bone should be evaluated for cortical erosion and focal osteopenia.

*Computed Tomography.* CT can assess periosteal reaction, cortical loss, and changes in bone marrow density [62].

*Magnetic Resonance Imaging.* The Infectious Disease Society of America guidelines recommends MRI to diagnose diabetic foot OM, even though bone biopsy with histopathology and microbiology is the “gold standard” [63,64]. Sensitivity and specificity of MRI for the early diagnosis of diabetic foot OM are 90% and 79%, respectively [65]. However, MRI displays low specificity and positive predictive value when there are non-infectious changes, especially in patients with previous foot surgery, trauma, or Charcot arthropathy [66,67].

On MRI diabetic foot OM is characterized by a high signal on T2-WI and STIR and signal on T1-WI [40,68]. However, when isolated, T2 hyperintensity or bone marrow edema is observed with no confluent intermediate T1 signal, and it is defined as “osteitis”. A T2 hyperintensity signal adjacent to a foot ulcer requires strict observation as it might become osteomyelitis in >50% of the patients [69].

*Nuclear Medicine.* Treglia et al. [70] observed that FDG PET and PET/CT have high specificity, with increased usefulness if combined with MRI. White blood cell PET/CT is the nuclear test of choice [71] whereas the role of FDG is still to be established for diabetic foot OM [72].

Limited three-dimensional resolution is a weakness of nuclear medicine. The vascular disease which predisposes patients to extremity OM can limit the distribution of isotopes distally [73].

## 7. Prosthetic Infections

Prosthetic joint infection (PJI) must be ruled out in any patient with a painful joint prosthesis [74]. Differential diagnosis between infection and aseptic loosening is essential because the treatment of these two complications is different.

Diagnostic criteria which include clinical examination, laboratory tests, and X-rays represent initial evaluation. C-reactive protein, (CRP), ESR, and leukocyte count are not adequately sensitive or specific. Articular aspiration, albeit specific, has a variable sensitivity [28].

The initial radiological assessment of implant infection is radiographic. X-rays and CT can exclude other potential causes of failed prosthesis (periprosthetic fracture, dislocation, breakage of prosthesis components, and aseptic loosening). They can also identify periosteal new bone formation, which is considered a specific feature of PJI, although with low sensitivity (16%) in early cases [75].

*Radiographs.* X-rays signs of PJI include sclerosis, periosteal reaction/cortical thickening, soft tissue gas, and component loosening. However, many of these can be seen also in aseptic loosenings. Thus, they are neither sensitive nor specific for PJI. Exuberant periosteal reaction and rapid radiographic progression are more suggestive of PJI [76]. Radiolucency along the metal-bone or cement bone interface that measures greater than 2 mm in width is another important abnormal finding [77].

*Ultrasonography.* Van Holsbeeck et al. [78] observed that US distention of the pseudocapsule greater than 3.2 mm was 100% sensitive and 74% specific for PJI. It was 100% specific for the diagnosis of PJI if combined with an extracapsular fluid collection. On the other hand, Weybright et al. [79] confirmed that although US is effective for the diagnosis of extracapsular fluid collections, it is not precise for diagnosing joint effusions. Moreover, the US can help in demonstrating fluid collection or sinus tracts in the soft tissues [80].

*Computed tomography.* CT is not normally useful for the diagnosis of PJI, since artifacts reduce image resolution [75,81]. It can detect focal and non-focal areas of periprosthetic osseous reabsorption. Periostitis was reported to be a specific (100%) sign of infection, even if not sensitive [75]. Isern-Kebschul et al. [82] proposed the assessment of multiple parameters on CT as a useful diagnostic method in patients with suggestive symptoms of complications after total hip arthroplasty.

*Magnetic Resonance Imaging.* MRI is costly and time-consuming compared with CT. It has limited usefulness to evaluate small periprosthetic osteolysis and the position of the prosthetic components. It is not useful to guide joint aspiration or tissue biopsy. Metal arthroplasty components distort the magnetic field, resulting in a well-known imaging artifact called magnetic susceptibility. This can limit visualization of the periprosthetic bones and soft tissues, which has historically limited MRI utility in the evaluation of these patients. However, numerous new MRI systems have been introduced recently to reduce metal artifacts and ameliorate the visualization of bones and soft tissues adjacent to a metal implant [83,84,85].

MRI features in PJI generally comprise pericapsular soft-tissue edema, extracapsular collections, bone destruction, reactive lymphadenopathy, and joint effusion with debris and thick hyperintense synovium formed of multiple layers. The existence of periosteal reaction, capsule edema, and intramuscular edema after hip arthroplasty at 1.5 T MRI with metal artifact reduction have a high accuracy to evaluate PJI [84]. However, one positive MRI sign, such as periosteal reaction, is not specific for hip PJI [86].

Some papers reported high sensitivity and specificity of lamellated hyperintense synovitis as an MRI characteristic of PJI in patients with knee [87,88] and hip [89] prosthesis. Lamellated hyperintense synovitis in an MRI image refers to the thickened synovial tissue nearby the joint in MRI, and “high signal” refers to the high signal in the T2WI sequence image. Albano et al. [86] suggested that the evaluation of lymph node size and number between the affected and unaffected sides may improve the diagnosis of PJI in THA.

*Nuclear Medicine.* The initial radionuclide test made is generally BS. It is mainly used to rule out PJI because of its high sensitivity, but it has low specificity (even lower −35%- in post-traumatic patients) [90]. In particular, the diagnostic reliability of BS is low in the first two years after prosthesis implantation. A positive BS does not confirm PJI, because it can also be positive because of another underlying bone disease or surgical intervention [91]. The diagnosis of PJI can be excepted with a negative BS. However, in the case of a positive BS, the addition of LLS significantly rises the diagnostic reliability for PJI, being useful in the differential diagnosis between PJI and reactive changes and/or aseptic loosening. In the case of a negative LLS, the probability of PJI is low [92].

PET/CT offers valuable information to evaluate a suspected PJI, regardless of which radiopharmaceutical is used [93,94]. It precisely localized abnormal white blood cells uptake, differentiating PJI and soft-tissue infection and providing information about the presence and the extent of infection. _18F_FDG PET/CT has a higher sensitivity (probably due to the subacute or chronic nature of most PJI, commonly involving monocytes and lymphocytes) but lower specificity than LLS [95,96]. For diagnosis, the site of the augmented uptake looks to be more important than its intensity (SUV), and uptake due to metallic artifacts should be taken into account [97]. Nonetheless, after surgery or trauma, _18_FDG-PET must be avoided for 3 to 6 months to decrease the risk of false positive results [98].

When infection is suspected, LLS with 99mTc-hexamethyl propylene amine oxime or exametazime is the nuclear exam of choice in the first years after prosthesis implantation or after a non-conclusive BS [99]. A study without leukocyte uptake supports the absence of infection; weak uptake which reduces over time suggests inflammation (or aseptic loosening of prosthesis), whereas a leukocyte uptake which progressively intensifies and/or extends suggests PJI.

## 8. Fracture Related Infection (FRI)

Fracture-related infection (FRI) is an infection that occurs in the presence of a fracture [100]. This includes early infection around fracture implants, infected non-unions, hematogenous infections arising after fracture healing, and infections in fractures with no internal fixation [101]. When FRI is suspected, imaging is necessary to evaluate fracture consolidation and implant stability, confirm the infection, and assess the extent of the infection with specific anatomical details for surgical planning [100].

*Radiographs.* Although plain radiograph shows low sensitivity and specificity for the diagnosis of FRI [102], it is the first investigation of choice to judge implant positioning, fracture reduction, and osseous healing [8,102]. X-rays are also important to assess the progress of fracture consolidation and disease evolution by follow-up examinations [103].

Similar to OM, signs of acute infection include soft tissue swelling, periosteal reaction, and intraosseous abscesses [8,102,103]. Chronic infectious features comprise a sequestrum, involucrum, and elevation of the periosteum [8,103], whereby all osseous changes are much better localized and earlier detected with CT [103,104].

*Computed tomography.* CT may also detect intra-medullar gas, which is considered a reliable sign of acute infection and allows a more detailed judgment of fracture consolidation [8,103].

*Magnetic Resonance Imaging.* MRI has an excellent sensitivity to detect FRI (82–100%) [104]. It identifies bone marrow edema as early as 1–2 days after onset of infection and soft tissue changes (such as abscesses, fistulae) [8]. With artifact reduction techniques, the interference of metal implants can be reduced to a minimum [105]. The downside of MRI is that its specificity is reduced probably because of its inability to differentiate between sterile inflammation, normal bone healing, and infected tissue (43–60%) [104].

*Nuclear Medicine.* Nuclear imaging is far more accurate in cases where it is important to distinguish infected from non-infected tissues. Three-phase BS is highly sensitive for detecting FRI (89–100%), but it has very low specificity (0–10%) [104], thus being obsolete for this indication.

For suspected FRI less than 2 years after fracture fixation, the nuclear methods of choice are the LLS [102,103,106], in which own white blood cells are labeled ex vivo with Indium-111 or Tc99m-HMPAO [102] and then re-injected. LLS + SPECT/CT is slightly more accurate (sensitivity 79–100% and specificity 97%) [91] than _18_FDG-PET/CT (sensitivity 88–89% and specificity 76–80%) [107]. However, although less accurate, _18_FDG-PET has major advantages over LLS in terms of lower complexity of the labeling procedure, the requirement for just one scan, rather than early and late phase scans (over 20 h), and its higher spatial resolution (3–4 mm vs. 8 mm) [106]. In addition, LLS is not appropriate in patients who have recently been on antibiotics [108].

Nonetheless, despite extensively available data on nuclear imaging in OM in general, there is a lack of studies exclusively focusing on FRI. Therefore, there is a need for future randomized controlled trials on optimal diagnostic strategies for FRI [109,110].

## 9. Spondylodiscitis

The majority of Infectious spondylodiscitis (SD) is the consequence of hematogenous seeding of the subchondral bone with extension to the intervertebral disc. It can also come from a prior operation or extension of an adjacent soft tissue infection [111]. The diagnosis of SD is often a challenge. Imaging is necessary for the diagnosis, localization of the infection and definition of its extension, identification of an appropriate region to perform a biopsy, assessment of neurological and infectious complications, and evaluation of response to treatment. Imaging assessment of patients with SD should include x-rays of the vertebral column and MRI with contrast medium administration [112].

*Radiographs.* On X-rays, the combination of rapid loss of intervertebral disc height and adjacent lysis of bone is evocative of an infection. Progressive destruction of the vertebral body and the intervertebral disc becomes evident with the further spread of infection, and the process soon contaminates the adjacent vertebra [5].

*Computed tomography.* CT-guided needle biopsy is essential for diagnosis confirmation and isolation of the responsible microorganism [113,114].

*Magnetic Resonance Imaging.* MRI is the most used procedure in SD, having a sensitivity higher than 90% [115]. Advantages include precise anatomical localization, early recognition of disc and bone destruction, the chance to perform multiplanar sequences, assessment of the bone marrow, and visualization of neural structures and soft tissues. However, it is limited by its low specificity because of false positive results in many cases such as in degenerative diseases, fractures by insufficiency, inflammatory processes, degenerative discal diseases with plate edema, vertebral amyloidosis, neuropathic arthropathy, and erosive intervertebral osteochondritis [116]. MRI is sensitive (up to 96%) but lacks specificity in the presence of fractures or spinal implants. Differential diagnosis from erosive osteochondritis is often difficult.

Vertebral osteomyelitis can be noticed early by MRI (before evident alterations emerge on X-rays) [117]. Involvement of two adjacent vertebrae may be observed 1 to 3 weeks before radiographic or CT signs of bone destruction.

Typical MRI findings of SD include (1) hypointense vertebral bodies and disc with loss of endplate definition in T1-weighted images, (2) hyperintense vertebral bodies and disc with loss of endplate definition in T2-weighted images or STIR images, and (3) Gadolinium enhancement of the vertebral body and disc. Imaging must comprise the whole spine to assess the extension of SD and to exclude any adjacent or skip lesions [118]. Early extension of inflammatory edema outside the limits of the vertebral bodies and the annulus fibrosus into the paravertebral fat causes low signal intensity on T1-weighted SE non-contrast images and hyperintense postcontrast and STIR images.

Depending on the infectious germ, the MRI features can be different. MRI can help in differentiating tubercular from pyogenic spondylodiscitis. The detection of an intact anterior meningo-vertebral ligament in presence of an epidural abscess is associated with tuberculous spondylodiscitis rather than pyogenic ones [119]. The presence of an almost undamaged vertebra with a homogeneous high signal on T2WI is an important MRI feature to distinguish Brucella SD from tubercular ones [120].

DWI of the spine showed a good association with the presence/absence of spinal infection and can be complementary to standard MRI with additional information. Apparent diffusion coefficients (ADC) are significantly reduced in patients with positive microbiological sampling compared to those with negative ones [121].

**Figure 5:**
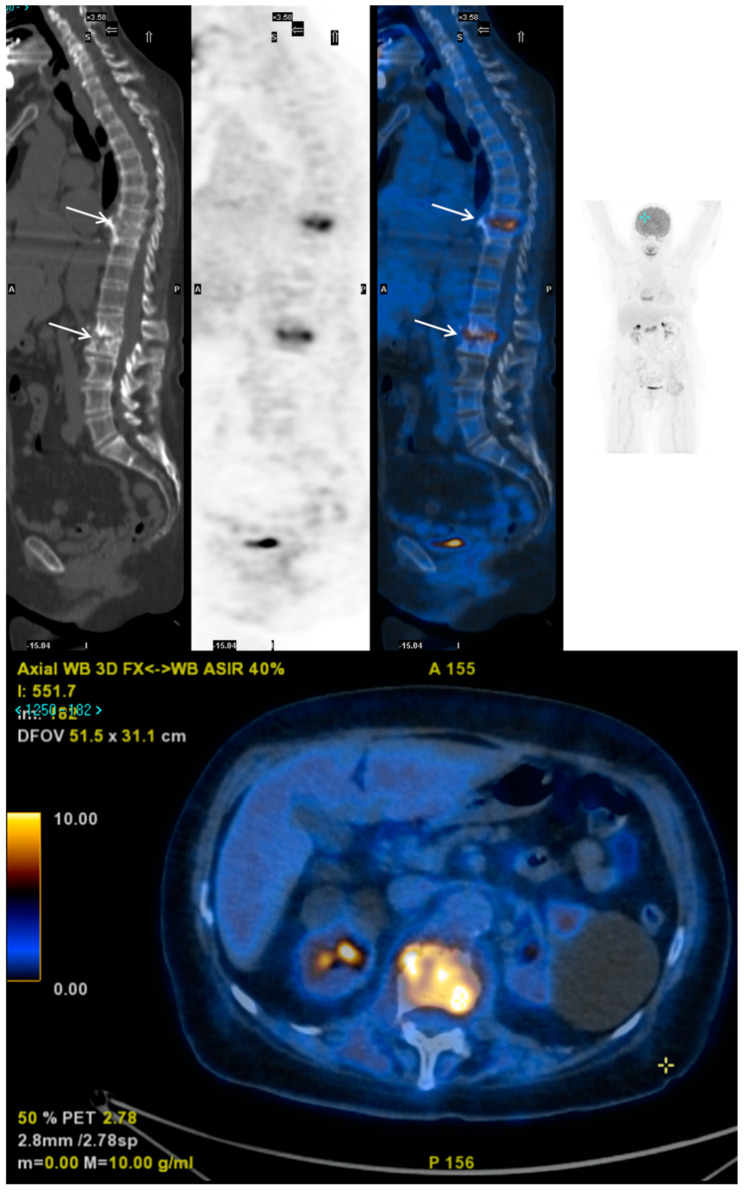
Pyogenic bifocal spondylodiscitis (T8-T9 and L1-L2) in a 70-year-old woman. FDG PET-CT showed increased SUV on both vertebral levels (arrows).

*Nuclear Medicine.* Radionuclide imaging is a helpful additional exam to MRI. It is commonly used as a screening test, but false-negative results happen. The test is of no use for detecting soft-tissue infections which can be associated or mimic spinal OM. Bone scintigraphy with _99_Tc or LLS is not regularly suggested because of low sensitivity and specificity. Gallium scans can play a role because if the result is negative, OM is improbable. Fuster et al. [122] compared the combination of BS + _67_Ga scan with SPECT/CT and _18F_FDG PET/CT to diagnose SD and confirmed that they both provided comparable information. A metanalysis by Prodromou et al. [123] showed a sensitivity of 97% and a specificity of 88%. The authors concluded that _18F_FDG PET/CT is an exceptional instrument in case of suspicion of SD. False positive results are commonly due to post-surgical alterations, spinal metastases, or metallic implants. A further advantage of _18F_FDG PET/CT is that it can be used both to assess response to therapy and to determine its length [124]. However, the test cannot discriminate infection from a tumor (Figure 5). Some studies revealed the superiority of _18F_FDG PET/CT over MRI, especially to discern degenerative changes from SD and to diagnose low-grade SD [125,126]. The use of _18F_FDG PET for diagnostic purposes is recommended in patients with a contraindication to MRI (due to metallic implants, pacemakers, or valve prostheses) or in nonconclusive MRI [127].

Newer tracers for BS such as indium-111 labeled (_111_In) biotin and streptavidin have been recently presented, with high sensitivity, specificity, and diagnostic accuracy for spinal infections [128]. Other new tracers include the technetium Tc-99m-ubiquitin-derived peptide which has a high affinity to areas with viable bacterial growth, in addition to radiolabeled antifungal tracers to distinguish fungal from bacterial infections [129].

## 10. Tuberculosis Arthritis

Tuberculous (TB) spondylitis (Pott’s disease) is the principal site of tuberculous bone involvement (approximately 50% of cases) [130]. It usually involves the thoracic and, less often, the lumbar vertebral column [131].

*Radiographs.* X-rays are of low sensitivity for the initial diagnosis of Pott’s disease. They identify vertebral involvement after at least 50% of a vertebra is damaged. Osteolytic lesions are more frequent than in pyogenic SD. Granulation tissue erodes and destroys cartilage and ultimately bone; areas of cartilage damage can be mixed with moderately normal zones.

*Computed tomography.* Thin-slice collimation spiral CT with multiplanar reconstruction can help in evaluating the damage of cancellous bone and deformity of the vertebral column in chronic cases (gibbus formation). Endplate destruction in tuberculous spondylitis often results in a more fragmented appearance than commonly observed with pyogenic organisms [132]. Spinal tuberculosis usually originates at the anteroinferior side of the vertebral body and extends to adjacent vertebrae along the anterior longitudinal ligament. Involvement of the posterior elements is rare but characteristic of TB, not found in pyogenic SD [133,134].

**Figure 6:**
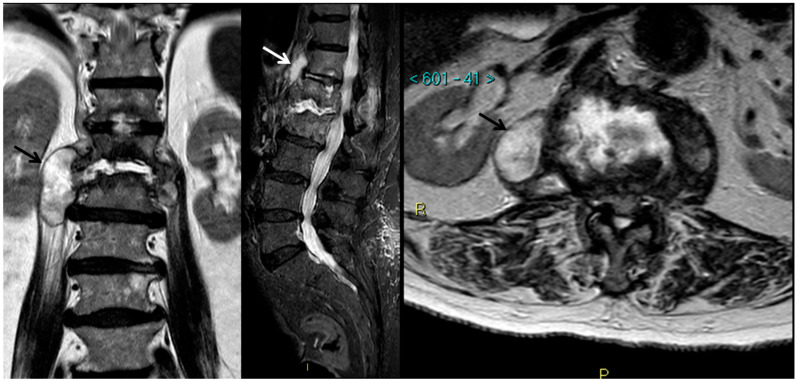
Tuberculous spondylodiscitis (T12-L2) in an 84-year-old woman. MRI detects bone and disks involvement together with several voluminous paravertebral abscesses (arrows).

*Magnetic Resonance Imaging* MRI is superior to CT in the evaluation and follow-up on spondylitis [131]. Bone marrow alterations are non-specific and include a patchy high signal on T2-weighted and a low signal on T1-weighted images (Figure 6) [135]. Pott’s disease has a minor amount of marrow edema than SD [136]. Hypointense T2-weighted image (secondary to areas of caseation) is associated with soft tissue abscesses [137]. Both involved vertebral disc and vertebral body have a similar signal intensity. However, disc involvement happens delayed than in SD, because of the lack of proteolytic destructive enzymes. T1-weighted images show intense enhancement after gadolinium administration.

TB spondylitis is strongly suggested in case of a slowly progressive vertebral process with preservation of intervertebral discs, the subligamentous spread of infection with erosion of anterior vertebral margins, large and calcified soft tissue abscesses, and the absence of severe bony eburnation [138].

## 11. Chronic Recurrent Multifocal Osteomyelitis

Chronic recurrent multifocal osteomyelitis (CRMO) is an autoimmune, non-infectious OM that usually occurs in children. It was also reported in adults, generally associated with synovitis, acne, pustulosis, hyperostosis, and osteitis [139]. It often affects the metaphysis of long bones, the pelvis, the spine, or the shoulder and clavicle [140,141].

CRMO remains a diagnosis of exclusion since there are no widely accepted diagnostic criteria and disease biomarkers. Imaging techniques are the mainstay for the diagnosis of CRMO [141].

Imaging findings of CRMO are analogous to hematogenous OM, with osteolysis surrounded by periostitis and soft tissue edema and subsequent progress of bony sclerosis. The presence of sequestra, sinus tracts, and soft tissue abscess is more suggestive of infective OM. Inflammatory bone lesions may be observed on x-rays as radiolucent, osteolytic, or sclerotic lesions, particularly in late stages [142]. Involvement of the medial portion of the clavicle and symmetric bilateral lesions in a patient without known malignancy is very suggestive of CRMO.

**Figure 7:**
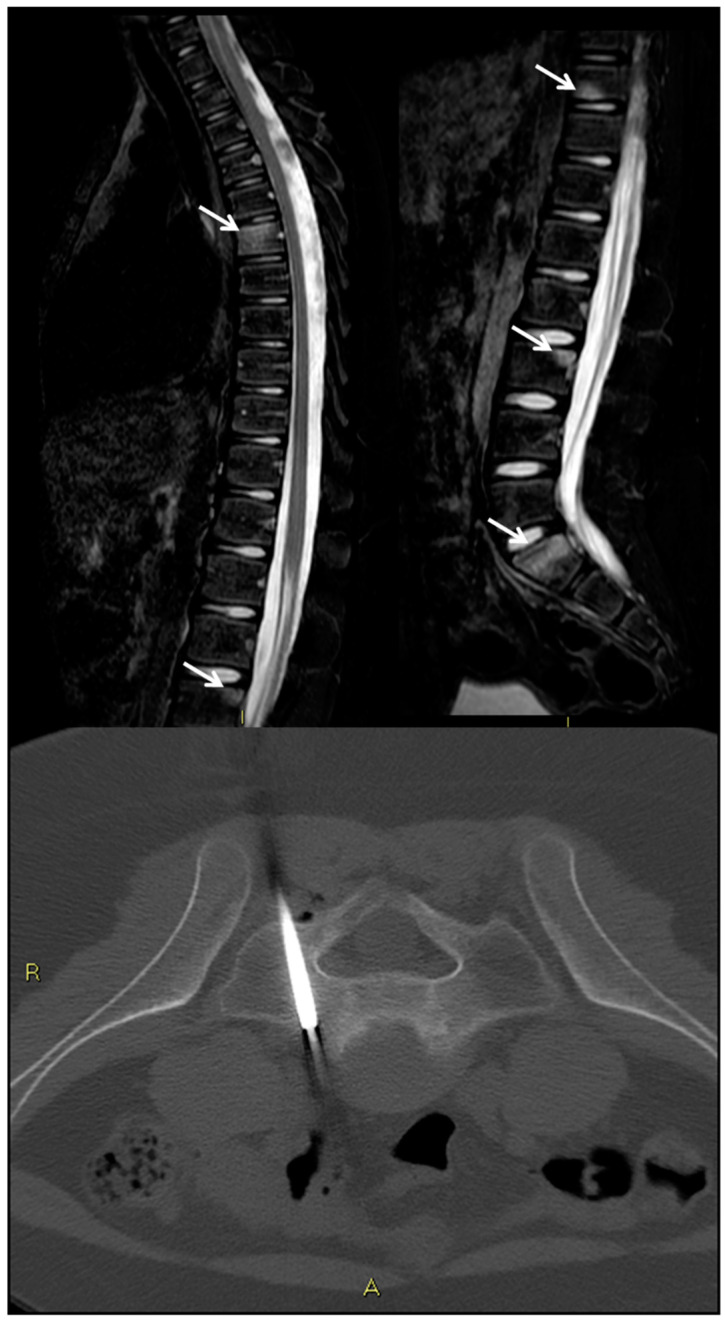
Chronic recurrent multifocal osteomyelitis (CRMO) in an 11-year-old boy. MRI shows several areas of bone edema in thoracic, lumbar, and sacral vertebral bodies (arrows). After a CT-guided bone biopsy of S1, the diagnosis of exclusion was CRMO.

MRI is highly sensitive in particular in the initial phases (Figure 7). MRI is very sensitive for the early findings of CRMO, but even with MRI, the findings of marrow edema on T2 or short-tau inversion recovery (STIR) sequences are not specific [139]. MRI is important for the assessment of disease activity during follow-up [143]. Strongly T2-weighted sequences and/or gadolinium-enhanced T1 sequences with fat saturation are helpful for the identification of inflammatory bone lesions and/or periosseous affections [144,145].

CRMO is generally multifocal, although they often present with just a single site of pain. Whole-body (WB) imaging can identify additional asymptomatic or minimally symptomatic lesions, aiding in reaching the diagnosis of CRMO [143,146,147]. In the case of children with CRMO, it is important to have a relatively short scan time to eliminate or at least minimize the need for sedation. A full-sequence WB-MRI may take 4–6 h, which is not realistically feasible in children. STIR sequences are relatively fast sequences that are sensitive to the marrow edema seen in CRMO [144]. Many CRMO WB-MRI imaging protocols include STIR sequences only, while others also include diffusion and/or T1-weighted imaging [148]. Compared to WB-MRI, bone scans require radiation and have decreased sensitivity, spatial resolution, and limited ability to evaluate physeal disease. Thus, WB-MRI is superior to bone scan in delineating the extent of disease.

## 12. Conclusions

We acknowledge that this is a narrative review of the literature, with inherent biases, including no critical appraisal of the quality of included studies in a systematic manner. Osteoarticular infection must be differentiated above all from malignant tumors but also from some benign tumors and pseudo-tumors. The clinical and laboratory panels sometimes cannot discriminate and the diagnosis can only be obtained on histological analysis. Thus, delays in treatment and inadequate management can frequently occur.

Bone and joint infections are a very heterogeneous group of diseases in terms of both affected site and severity. In general, conventional radiographs of the affected site should be performed as the first imaging examination (Table 1).

Second-line tests should be decided on a case-by-case basis by the MDT, according to clinical suspicion, type of BJI, site affected, and patient’s characteristics, to reach an accurate diagnosis as soon as possible. Thus, these cases should be referred to multispecialty centers with all diagnostic tools. Recently, Sconfienza et al. proposed a flowchart that may guide the diagnosis of osteomyelitis and PJI [149].

New technologies such as PET/MRI are strongly emerging research platforms in imaging science [150] which might help in the diagnosis of bone infections. However, hybrid PET/MRI scanners are very expensive. Therefore, it seems preferable to use MRI as a primary imaging tool for uncomplicated unifocal cases, whereas in cases with (possible) multifocal disease or a contraindication for MRI, PET would be preferred.

Severe morbidity or mortality have been reported in BJI such as acute paraplegia in spine infections [151,152], severe and irreversible joint destruction and even death in septic arthritis [153], and sepsis and death in PJI [154].

However, no previous series reported on the possible consequences if the diagnosis is established late and the treatment is inadequate. However, the experience of the COVID-19 pandemic recently suggested that a delayed diagnostic process of disseminated invasive infections can increase the risk of fatal consequences, in particular in frail patients [155].

Therefore, an accurate and prompt diagnosis requires a high index of suspicion followed by the combination of adequate surgical and conservative treatment to prevent severe morbidity and decrease the risk of mortality.

## Figures and Tables

**Table 1:** A multidisciplinary team (MDT) evaluation which includes radiologist, orthopedic surgeon, infectious disease specialist, and microbiologist is essential for a correct diagnosis of infection.

	X-Ray	Ultrasonography	CT-Scan	MRI	Nuclear Medicine
**Acute Osteomyelitis**	low sensitivity during the first 10–14 days cortical bone destruction, marrow lucency periosteal reaction soft tissues alterations	juxtacortical swelling of soft tissues periosteal elevation or thickening possible abscess useful in US-guided biopsy	cortical erosion foci of gas soft tissue alterations sinus tracts	highly sensitive in the first 3–5 days medullary edema and exudates zones of necrosis soft tissue alterations/abscess	3-phase 99mTc BS: high negative predictive value LLS + SPECT/CT: method of choice in patients with a recent fracture or recent surgery 18FGD PET/CT: useful in multifocal osteomyelitis and differential diagnosis with tumors 18F-NaF PET/CT recently proposed
**Septic Arthritis**	bone erosions joint space loss periarticular osteopenia soft tissue swelling acute OM signs on both sides of the joint	joint effusion: high sensitivity, low specificity power doppler: synovial and soft tissue hyperemia useful in US-guided joint aspiration	joint effusion acute OM signs on both sides of the joint	joint effusion enhancing synovitis cartilage thinning periarticular soft tissue edema subperiosteal fluid collection	3-phase 99mTc BS: useful in differentiate OM from soft-tissue infection and in multifocal joint infections 18FDG PET: low specificity
**Chronic Osteomyelitis**	sclerosis and cortical thickening adjacent to lytic zones within the marrow	useful in case of recrudescence with acute OM signs	sclerosis and cortical thickening invasion of the medullary cavity sequestrum useful in CT-guided biopsy	sequestrum cloaca periostitis fibrovascular scar useful in differentiate acute from chronic OM	18FDG PET/CT: high sensitivity and specificity
**Brodie’s abscess**	usually, lytic unicameral or multiloculated lesion with a sclerotic rim	not routinary used in diagnosis	lytic lesion with a sclerotic rim well-circumscribed periosteal reaction useful in CT-guided biopsy	“target sign” peripheral ring contrast enhancement	scintigraphy generally positive 18FDG PET: unclear role
**Diabetic foot osteomyelitis**	foci of air cortical erosion focal osteopenia	not routinary used in diagnosis	periosteal reaction cortical erosion cortical loss changes in bone marrow density	variable acute and chronic OM signs	WBC PET/CT useful in diagnosis
**Prosthetic Infections**	sclerosis periosteal reaction cortical thickening soft tissue gas component loosening	distention of the pseudocapsule extracapsular fluid collection sinus tracts useful in US-guided joint aspiration	focal and non-focal areas of periprosthetic osseous reabsorption signs of periostitis and cortical alterations soft tissue gas	pericapsular soft tissue edema extracapsular collections bone destruction reactive lymphadenopathy, joint effusion thick or lamellated synovium	LLS + SPECT/CT: method of choice in patients with a recent fracture or recent surgery 18FDG-PET/CT: higher sensitivity but lower specificity than LLS, must be avoided for 3 to 6 months after surgery or trauma
**Fracture related infection**	low sensitivity and specificity eventually non-union eventually hardware failure eventually acute or chronic OM signs	not routinary used in diagnosis eventually acute or chronic OM signs	eventually non-union eventually hardware failure eventually acute or chronic OM signs	eventually acute or chronic OM signs	3-phase 99mTc BS: high sensitivity, low specificity LLS + SPECT/CT: method of choice for diagnosis 18FDG-PET/CT: high sensitivity and specificity, simpler method, useful in patients on antibiotic therapy
**Spondylodiscitis**	low sensitivity vertebral body deformity	not routinary used in diagnosis	vertebral body deformity endplate destruction useful in CT-guided biopsy	most used imaging technique high sensitivity, low specificity useful from 1 to 3 weeks before radiographic or CT signs T1-WI hypointense/T2-WI hyperintense vertebral bodies and disc loss of endplate definition high contrast enhancement	3-phase 99mTc BS and LLS: low sensitivity and specificity 67Ga SPECT/TC and 18FDG PET: high sensitivity and specificity new tracers for PET may increase sensitivity and specificity ∙
**Tuberculosis arthritis**	low sensitivity vertebral body deformity vertebral osteolytic lesions are more frequent than in pyogenic SD	not routinary used in diagnosis	vertebral body deformity often involvement of antero-inferior side of the vertebra posterior involvement more frequent than in pyogenic SD endplate destruction useful in CT-guided biopsy	useful for follow-up lower marrow edema than pyogenic SD areas of caseation intense contrast enhancement large and calcified soft tissue abscesses no bony eburnation	not able to distinguish between pyogenic and non-pyogenic infection
**Chronic recurrent multifocal osteomyelitis**	X-ray, US, CT-scan are analogous to infective OM sequestra, sinus tracts, abscess are less frequent often symmetrical distribution (clavicles often involved) more frequent in children			whole body STIR sequences useful in diagnosis	scintigraphy less sensitive/specific than whole body MRI 18FDG PET: unclear role

## Data Availability

Not applicable.
